# Disease Modeling and Disease Gene Discovery in Cardiomyopathies: A Molecular Study of Induced Pluripotent Stem Cell Generated Cardiomyocytes

**DOI:** 10.3390/ijms22073311

**Published:** 2021-03-24

**Authors:** Satish Kumar, Joanne E. Curran, Kashish Kumar, Erica DeLeon, Ana C. Leandro, Juan Peralta, Sarah Williams-Blangero, John Blangero

**Affiliations:** 1 Department of Human Genetics and South Texas Diabetes and Obesity Institute, University of Texas Rio Grande Valley School of Medicine, McAllen, TX 78504, USA; kashish.kumar01@utrgv.edu (K.K.); erica.deleon02@utrgv.edu (E.D.); sarah.williams-blangero@utrgv.edu (S.W.-B.); 2 Department of Human Genetics and South Texas Diabetes and Obesity Institute, University of Texas Rio Grande Valley School of Medicine, Brownsville, TX 78520, USA; joanne.curran@utrgv.edu (J.E.C.); ana.leandro@utrgv.edu (A.C.L.); juan.peralta@utrgv.edu (J.P.); john.blangero@utrgv.edu (J.B.)

**Keywords:** human, induced pluripotent stem cell, cardiomyocytes, cardiomyopathies, genome-wide mRNA sequencing

## Abstract

The in vitro modeling of cardiac development and cardiomyopathies in human induced pluripotent stem cell (iPSC)-derived cardiomyocytes (CMs) provides opportunities to aid the discovery of genetic, molecular, and developmental changes that are causal to, or influence, cardiomyopathies and related diseases. To better understand the functional and disease modeling potential of iPSC-differentiated CMs and to provide a proof of principle for large, epidemiological-scale disease gene discovery approaches into cardiomyopathies, well-characterized CMs, generated from validated iPSCs of 12 individuals who belong to four sibships, and one of whom reported a major adverse cardiac event (MACE), were analyzed by genome-wide mRNA sequencing. The generated CMs expressed CM-specific genes and were highly concordant in their total expressed transcriptome across the 12 samples (correlation coefficient at 95% CI =0.92 ± 0.02). The functional annotation and enrichment analysis of the 2116 genes that were significantly upregulated in CMs suggest that generated CMs have a transcriptomic and functional profile of immature atrial-like CMs; however, the CMs-upregulated transcriptome also showed high overlap and significant enrichment in primary cardiomyocyte (*p*-value = 4.36 × 10^−9^), primary heart tissue (*p*-value = 1.37 × 10^−41^) and cardiomyopathy (*p*-value = 1.13 × 10^−21^) associated gene sets. Modeling the effect of MACE in the generated CMs-upregulated transcriptome identified gene expression phenotypes consistent with the predisposition of the MACE-affected sibship to arrhythmia, prothrombotic, and atherosclerosis risk.

## 1. Introduction

Cardiomyopathies are a heterogeneous group of diseases caused by pathological alterations in myocardial structure and function and often progress into heart failure (HF) and death. The myocardial alterations can be either solely or predominantly confined to the heart, in which they are generally classified as primary cardiomyopathies or as part of more generalized systemic (multiorgan) disorders that are classified as secondary cardiomyopathies. Both primary and secondary cardiomyopathies may result from genetic predisposition and/or acquired non-genetic factors including exposure to environmental toxins, chemotherapeutic drugs, or infectious agents [1]. Based on the structural and functional changes that take place at the whole-organ level, cardiomyopathies are classically divided into (i) dilated cardiomyopathy (DCM), defined as the dilatation of the left or both ventricles as a consequence of impaired myocardial contractility in the absence of abnormal overload and/or ischemic heart disease [2]; (ii) hypertrophic cardiomyopathy (HCM), characterized by inappropriate myocardial hypertrophy that develops in the absence of pressure overload or infiltration [3]; (iii) arrhythmogenic cardiomyopathy (AC), characterized by progressive fibrofatty replacement of the ventricular myocardium leading to arrhythmia, HF, and sudden cardiac death [4]; and (iv) restrictive cardiomyopathy (RCM), characterized by impaired ventricular filling and diastolic function with relatively normal ventricular wall thickness and systolic function [5]. This traditional classification has continuing relevance for clinical diagnosis and management; however, there is extensive overlap between these phenotypes. For example, HCM or AC may progress into a dilated ventricle with systolic dysfunction and hence the appearance of DCM [3,6]. Furthermore, DCM encompasses a wide range of genetic and acquired disorders having a greater or lesser impact in different patients and over the course of a patient’s life [7,8]. For example, early DCM stages may present intermediate phenotypes that do not meet the classical definition of the disease. Therefore, cellular and molecular phenotypes, and diagnostic criteria that are more precise and robust, are needed for early detection and interventions in cardiomyopathies.

The monogenic form of inherited cardiomyopathies represents a small proportion of all HF cases; however, this varies significantly by age and population studied [9,10]. Susceptibility to HF was identified as a complex heritable trait in the Framingham Offspring cohort, even after adjusting for comorbidities like myocardial infarction, diabetes mellitus, and hypertension [11]. Furthermore, due to the heterogeneous nature of the disease phenotype and disease penetrance, precise data showing prevalence of different cardiomyopathies in the general population are lacking. For example, overall prevalence of idiopathic DCM is estimated at around 1 case per 2500 individuals; however, a frequency ten times higher than this estimate was reported in a previous publication [7], suggesting a large proportion of this debilitating disease, and its predisposition goes undetected or is detected at a very late stage.

Early breakthroughs with in vitro modeling of cardiac development and cardiomyopathies in human embryonic stem cell (ESC)-derived and induced pluripotent stem cell (iPSC)-derived cardiomyocytes (CMs) have been very promising [12,13,14,15]. However, an in vitro iPSC-derived cardiomyocyte model of cardiomyopathies and associated heart diseases, whose production is scalable to a larger sample size, is essential to aid the discovery of molecular and developmental changes that take place in cardiomyopathies and the genes or genetic variants influencing these disease phenotypes to develop new diagnostics and therapeutics.

In this study, we have generated well-characterized cardiomyocytes from lymphoblastoid cell line (LCL) reprogrammed iPSC lines of 12 participants of our San Antonio Mexican American Family Study (SAMAFS), who belong to four sibships ranging from 2 to 4 siblings per sibship. The validated iPSCs and their differentiated CMs were analyzed using genome-wide RNA sequencing based transcriptomic and functional annotation analyses to better understand the functional characteristics and disease modeling potential of the generated CMs.

The existing rich bio-resource of immortalized LCL repositories generated from a wide array of patients in genetic and epidemiological studies worldwide, many of them with extensive genotypic, genomic, and phenotypic data already existing, provides a great opportunity to reprogram iPSCs from any of these LCL donors in the context of their own genetic identity for disease modeling and disease gene identification in cardiomyopathies [16,17,18].

## 2. Results

### 2.1. Differentiation and Lineage-Specific Functional Characteristics of Generated CMs

**Figure 1:**
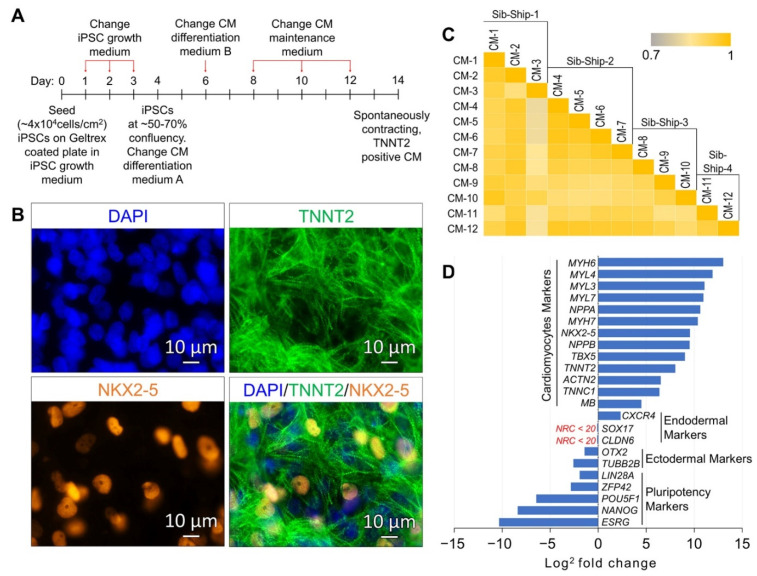
Characterization of induced pluripotent stem cell (iPSC)-generated cardiomyocytes (CMs) by ICC and gene expression analysis. (**A**) Schematic diagram outlining the protocol of iPSC to CM differentiation. (**B**) A representative ICC image panel of iPSC-generated CMs showing expression of the CM markers NKX2-5 and TNNT2. (**C**) Correlation coefficient (r^2^) plot based on the 12,280 genes found expressed (NRC ≥ 20) in 12 iPSC-generated CMs. (**D**) Gene expression plot showing average differential expression of cardiomyocytes, pluripotency, ectodermal, and endodermal markers between 12 iPSCs and their generated 12 CM lines.

The CM differentiation of validated iPSC lines, reprogrammed using LCLs of 12 SAMAFS participants and the method described in our previous publications [17,18] and detailed in Figure S1, was induced using the Gibco PSC Cardiomyocyte Differentiation Kit (Thermo Fisher Scientific, Waltham, MA, USA), and the method is summarized in the schematic presented in Figure 1A. The differentiation kit consists of a set of serum-free and xeno-free media that enable efficient differentiation of human iPSCs to spontaneously contracting functional cardiomyocytes. On day 14 of the differentiation, spontaneously contracting CMs were characterized by immunocytochemistry (ICC) analysis of cardiac mesoderm marker NKX2-5, mature cardiomyocyte marker TNNT2, and by genome-wide RNA sequencing-based gene expression analysis. The CMs differentiated from all 12 samples expressed the cardiomyocyte markers NKX2-5 and TNNT2 (Figure 1B and Figure S2). Based on the criteria normalized read count (NRC) ≥ 20 in all 12 CM samples, a total of 12,280 genes were found expressed. The average correlation coefficient at 95% CI between 12 CM samples, calculated based on all expressed genes, was 0.92 ± 0.02, suggesting a uniform differentiation of generated CMs across 12 samples (Figure 1C). The expressions of the well-known CMs genes/markers *MYH6*, *MYL4*, *MYL3*, *MYL7*, *NPPA, NPPB*, *MYH7*, *NKX2-5*, *TBX5*, *TNNT2*, *ACTN2*, *TNNC1* and *MB* were significantly up-regulated across all 12 CM samples (FDR corrected *p*-value ≤ 0.05 and fold change absolute (FC-abs) ≥ 2.0); whereas the expressions of pluripotency and ectodermal markers were significantly down-regulated. The endodermal markers *SOX17* and *CLDN6* were below the expression threshold of NRC ≥ 20 (Figure 1D and Table S1). The expression of another endodermal marker *CXCR4* was up-regulated (FC = 5.0) in the iPSC-generated CMs. *CXCR4* is expressed in native cardiomyocytes and plays an important role during development and in adult tissue homeostasis [19,20,21].

### 2.2. CM Subtypes and Maturation State

**Figure 2:**
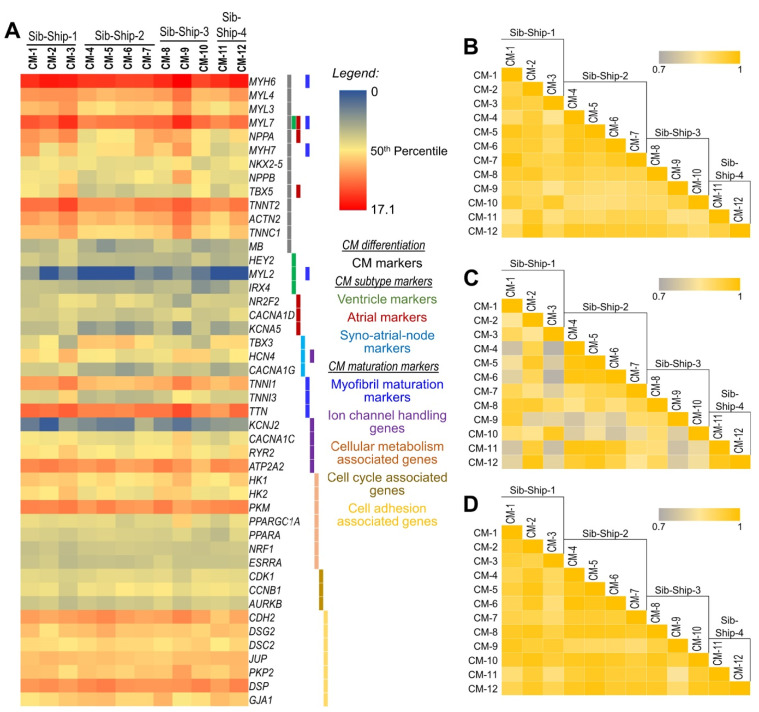
Expression analysis of CM-specific, CM-subtype-specific, and CM maturity-associated genes to assess uniform CM population across 12 CM samples. (**A**) Heat map showing normalized (log^2^) expression of CM-specific, CM-subtype-specific, and CM maturity-associated genes in 12 iPSC-generated CM lines. (**B**–**D**) Correlation coefficient (r^2^) plots based on (**B**) CM-specific, (**C**) CM-subtype-specific, and (**D**) CM maturity-associated genes in 12 iPSC-generated CM lines, respectively.

Though some breakthroughs in atrial and ventricular specification of generated CMs have been achieved [22], most currently available differentiation methods produce a heterogeneous population of CM subtypes, including ventricular-, atrial-, and pacemaker-like CMs. Any heterogeneity in differentiated CM populations, which may include CM and nonCM cells, CM subtypes, and/or maturation state across different samples, may confound disease modeling experiments particularly in a large, epidemiological-scale disease gene discovery approach. Assuming that samples with a highly enriched and uniform CM population will show significantly high correlation in the expression of CM-specific marker genes, we performed sample correlation analysis using the quantitative expression of 13 CMs-specific markers (Figure 2A). The average correlation coefficient at 95% CI between 12 CM samples, calculated based on the expression of 13 specific CM genes, was 0.94 ± 0.02, which suggests a uniform, highly enriched CM population across the 12 samples (Figure 2B). Second, we evaluated the concordance of CM subtypes (i.e., ventricular-, atrial-, and sinoatrial-node-like CMs) in the differentiated CM population across the 12 samples using quantitative expression of CM subtype specific genes (Figure 2A). The ventricle-specific gene *MYL2* was below the expression threshold (NRC ≥ 20), and *HEY2,* though expressed in all samples, did not change significantly between iPSCs and differentiated CMs. The expressions of other ventricle-associated genes *IRX4* and *MYH7* were significantly upregulated (FC = 20.4 and 1331.1, respectively). The expressions of atrial-specific genes (*NR2F2*, *TBX5*, *NPPA*, *MYL7*, *CACNA1D,* and *KCNA5*) and sino-atrial-node-associated genes (*TBX3*, *HCN4,* and *CACNA1G*) were also significantly upregulated (FDR corrected *p*-value ≤ 0.05 and fold change FC ≥ 2.0). These gene expression profiles suggest that the majority of the CM population is of the atrial subtype (Figure 2A). To assess the CM subtype concordance across the 12 samples, a sample correlation analysis using these CM subtype specific genes was performed, and the average correlation coefficient at 95% CI between the 12 CM samples was 0.89 ± 0.04, suggesting concordant CM subtypes across these samples (Figure 2C). Thirdly, the maturation state of the generated CMs across the 12 samples was assessed using quantitative expression of genes associated with biological processes involved in CM maturation, that is (1) myofibril maturation marked by isoform switching of several sarcomere components such as *MYH6* to *MYH7*, *TNNI1* to *TNNI3*, *MYL7* to *MYL2*, and *TTN-N2BA* to *TTN-N2B*; (2) changes in the expression of ion channel handling genes (*KCNJ2*, *HCN4*, *CACNA1C*, *RYR2*, and *ATP2A2*); (3) changes in the genes involved in cellular metabolism (*HOOK1*, *PKM*, *PPARGC1A*, *PPARA,* and *ESRRA*); and (4) changes in the cell cycle and cell adhesion associated genes (*CDK1*, *CCNB1*, *AURKB*, *CDH2*, *DSG2*, *DSC2*, *JUP*, *PKP2*, *DSP,* and *GJA1*). A sample correlation analysis using these CM maturity-associated genes (correlation coefficient at 95% CI 0.95 ± 0.02) suggest a uniform CM maturation state across the 12 samples (Figure 2A,D).

### 2.3. Differentially Expressed (DE) Genes

To identify the unique transcriptomic signature of the generated CMs and the mechanistic gene expression changes that occurred during the differentiation of validated iPSCs into CMs, we performed a genome-wide differential gene expression analysis between the 12 iPSCs and their differentiated CMs.

A total of 271 and 167 million mRNA 50bp single-end reads were obtained for 12 iPSC lines and their differentiated CMs, respectively.

**Figure 3:**
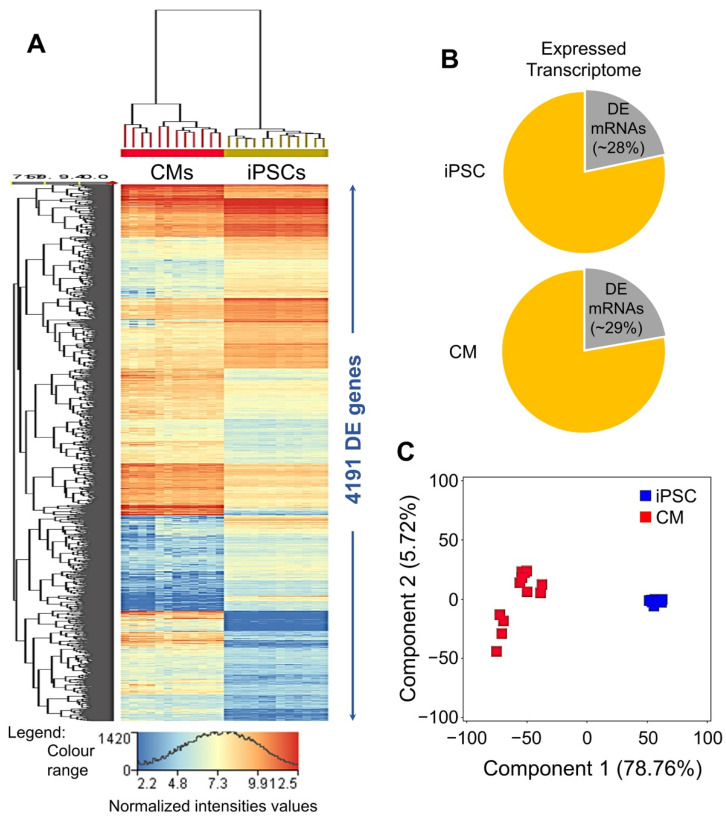
Genome-wide differential gene expression analysis of 12 iPSCs and their differentiated 12 CM lines. (**A**) Heat map of differentially expressed (DE) genes between 12 iPSCs and their 12 differentiated CM lines. (**B**) Pie graph showing average DE vs. total expressed transcriptome in 12 iPSCs and their differentiated CM lines. (**C**) Principal component analysis (PCA) of genes found DE between 12 iPSCs and their 12 differentiated CM lines.

The 13,460 genes having an NRC ≥ 20.0 in all 12 iPSC and/or 12 CM samples were considered expressed and included in the differential gene expression analysis. Following the criteria moderated *t* statistics FDR-corrected *p*-value ≤ 0.05 and FC-abs ≥ 2.0, 4191 genes were found significantly DE between iPSCs and their differentiated CMs (Table S1) and accounted for nearly 29% of the CM’s and 28% of the iPSC’s expressed transcriptome (Figure 3A,B). About 79% of the observed variance in DE genes can be attributed to the iPSC-to-CM cellular transition (Figure 3C).

### 2.4. Functional Annotation Analysis of DE Genes

A total of 2075 DE genes were significantly down regulated in differentiated CMs and included stemness, pluripotency and self-renewal associated transcription factors (*POU5F1*, *NANOG*, *SOX2*, *FOXD3*, *ZFP42,* and *NR6A1*), signaling molecules (*GABRB3*, *GAL*, *GRB7*, *IFITM1*, *KIT*, *SFRP2*, and *TDGF1*), and other human iPSC/ESC-specific genes (*CD9*, *DIAPH2*, *DNMT3B*, *LIN28A*, *PODXL*, *TERT*, *ESRG,* and *GJA1*). The functional annotation analysis performed using the Ingenuity Pathway Analysis (IPA) platform (QIAGEN Digital Insights, Redwood City, CA, USA) showed significantly high enrichment of the 2075 down-regulated genes in cell death and survival (685 mRNAs; FDR-adjusted *p*-value range: 6.74 × 10^−23^–3.35 × 10^−7^), cell cycle (355 mRNAs; FDR-adjusted *p*-value range: 8.94 × 10^−20^–3.71 × 10^−7^), cellular assembly and organization (391 mRNAs; FDR-adjusted *p*-value range: 8.94 × 10^−20^–3.76 × 10^−7^), and DNA replication, recombination, and repair (278 mRNAs; FDR-adjusted *p*-value range: 8.94 × 10^−20^–3.96 × 10^−7^) cellular functions. The downstream effects analysis as implemented in IPA predicted significant deactivation (activation *z*-score *≤* −2.0) of cellular functions and processes associated with iPSC/ESC self-renewal (Table S2).

A total of 2116 DE genes were significantly upregulated in differentiated CMs. Using IPA, these genes showed significantly high enrichment in cardiovascular system development and functions (571 mRNAs; FDR-adjusted *p*-value range: 1.75 × 10^−66^–1.61 × 10^−14^), and IPA predicted significant activation of the functions associated with development of the human heart and cardiovascular system (Table S3). Additionally, we performed enrichment analysis of the upregulated genes into gene ontology (GO) biological processes. The top 10 significantly enriched GO biological processes are presented in Table 1, and results further validate the developmental and functional profiles of generated CMs as determined in the IPA analysis.

For more detailed characterization and to elucidate the disease modeling potential of iPSC-generated CMs, we performed enrichment analysis of the 2116 genes upregulated in CMs into several cell-type-specific and disease-specific gene sets as implemented in Enrichr, a gene list enrichment analysis web tool [23,24].

The enrichment analysis of the CM-upregulated genes in the specific tissue/cell type gene sets presented in Table 2 shows significant enrichment of our iPSC-generated cardiomyocyte-upregulated genes in the primary cardiomyocytes and primary human heart tissue gene sets.

The upregulated CM genes also showed significant enrichment in ClinVar (2019) gene sets for human cardiomyopathies. The top five highly enriched gene sets are listed in Table 3 and show that the iPSC-generated CMs have the potential for in vitro modeling of human cardiomyopathies including COVID-19-induced cytopathic effects on the human heart.

### 2.5. Modeling the Effect of Major Adverse Cardiac Event (MACE) in Familial Sample

**Figure 4:**
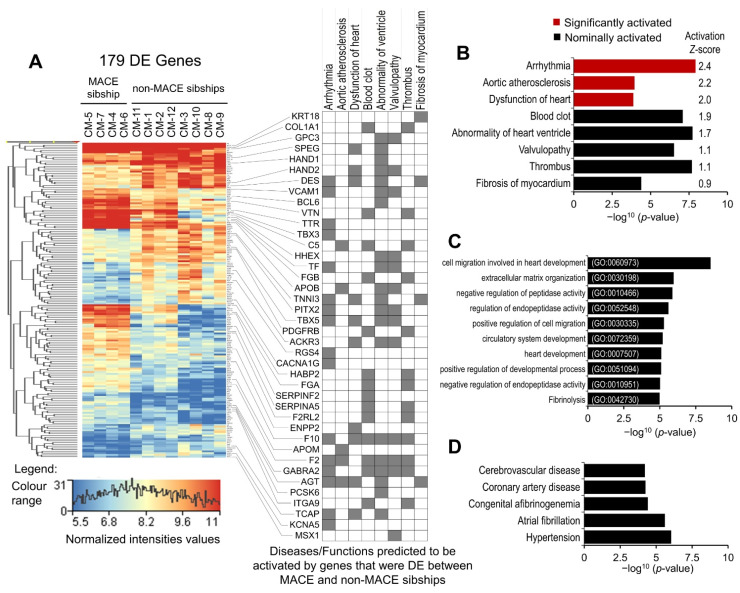
Differential gene expression analysis of CMs-upregulated transcriptome between MACE-affected (*n* = 4) and unaffected sibships (*n* = 8). (**A**) Heat map of 179 DE genes and disease annotation grid showing enrichment of DE genes in diseases that were predicted to be activated in IPA analysis. (**B**) Enrichment *p*-values and activation *z*-score of three significantly activated and five nominally activated diseases, predicted in IPA disease enrichment analysis. (**C**) Top 10 significantly enriched GO biological processes in MACE-associated DE genes. (**D**) Top 5 significantly enriched disease conditions identified in Jensen diseases data base enrichment analysis of MACE-associated DE genes.

As indicated in the introduction section, the 12 SAMAFS participants whose iPSC lines were used to differentiate CMs belonged to 4 sibships of size 4, 3, 3, and 2 siblings, respectively, and 1 participant in the sibship of 4 reported MACE. Considering that the 2116 significantly up-regulated genes are the most functionally relevant gene set of the generated CMs, we performed differential gene expression of the CM-upregulated genes between the sibship with one MACE-affected sibling (*n* = 4) and the three unaffected sibships (*n* = 8). A total of 179 genes were found to be significantly DE (moderated *t* statistics *p*-value ≤ 0.05 and FC-abs ≥ 2.0) between the affected and unaffected sibships (Table S4). The disease and function annotation analysis of the 179 DE genes in IPA showed significant enrichment and predicted activation of cardiovascular disease associated conditions. Arrhythmia, aortic atherosclerosis, and dysfunction of heart were predicted to be significantly (activation *z*-score ≥ 2.0) activated. Blood clot, abnormality of ventricle, valvulopathy, thrombus, and fibrosis of myocardium were predicted to be nominally (2.0 > activation *z*-score ≥ 0.9) activated (Figure 4A,B). To validate our IPA enrichment analysis results, we performed enrichment analysis of the 179 DE genes in GO biological processes (2018) and Jensen diseases, a disease gene association database mined from the literature [25]. The top 10 highly enriched GO biological processes presented in Figure 4C included processes involved in the development of heart and vascular tissue, negative regulation of peptidase activity, and fibrinolysis. The top five enriched diseases included cerebrovascular disease, coronary artery disease, congenital afibrinogenemia, atrial fibrillation, and hypertension (Figure 4D). These results suggest that coexistence of atrial fibrillation/arrhythmia, prothrombotic and atherosclerosis risk may be causal to or associated with MACE in the affected participant, and the other three members of the sibship may have a higher risk of heart disease and/or MACE. These results also show that our iPSC-generated CMs are a relevant cell model for investigating cardiovascular disease and cardiomyopathies.

## 3. Discussion

The in vitro generation of CMs by differentiating human ESCs and iPSCs provides opportunities for in vitro modeling of cardiomyopathies and associated cardiac diseases for disease gene discovery. Several challenges such as protocols for derivation of highly enriched differentiated CM populations, derivation of CMs that are specific to heart chambers, and maturation of generated CMs have been the focus of many studies and review articles [22,26,27,28,29,30]. While overcoming these challenges is essential in achieving critical properties of electrical excitability, electrical connectivity, calcium handling, and motor protein functions in generated CMs for regenerative medicine and some aspects of drug discovery and disease gene validation strategies, for a large, epidemiological-scale disease gene discovery approach, it is critical that the production of iPSC-differentiated CMs is scalable to a larger sample size and that the generated CMs are comparable in enrichment, CM subtypes, and maturation properties across samples. In this study, we have shown robust differentiation of well-characterized CMs (Figure 1) in a monolayer culture using iPSCs generated from cryopreserved LCLs of 12 participants of our SAMAFS and a commercially available Gibco PSC Cardiomyocyte Differentiation Kit (Thermo Fisher Scientific, Waltham, MA, USA). We performed genome-wide transcriptomic analysis to evaluate the differentiated CM characteristics across 12 samples and the disease modeling potential.

### 3.1. Characterization and Disease Modeling Potential of Generated CMs

The generated CMs showed very high concordance across the 12 samples in the CM’s total expressed transcriptome (average correlation coefficient at 95% CI = 0.92 ± 0.02) as well as showing a gene expression profile (Figure 1D) very similar to previously published gene expression profiles of highly enriched, iPSC-differentiated CMs [31,32,33]. The CMs-upregulated transcriptome (the 2116 genes significantly upregulated in CMs) and expressed transcriptome (the 12,280 genes having NRC ≥ 20 in all 12 CM samples) showed ~25% and ~65% overlap with upregulated genes in GTEx human heart tissue samples, respectively. Additionally, the CMs-upregulated transcriptome and expressed transcriptome showed 22.7% and 68.9% overlap with the Cardiac-Myocyte upregulated gene set from the BioGPS Human Gene Atlas [34]. These results and the enrichment analysis of the CMs-upregulated transcriptome into GO biological processes and human gene atlas and GTEx tissue samples upregulated gene sets presented in Table 1 and Table 2 suggest that our iPSC-generated CMs have a gene expression profile similar to primary human heart tissue and cardiomyocytes.

Enrichment analysis of the CM-upregulated transcriptome in GO biological processes shows significant enrichment and ~70% and ~65% gene overlap with ventricular cardiac muscle tissue morphogenesis (GO:0055010) and cardiac atrium morphogenesis (GO:0003209) biological processes, respectively (Table 1). Therefore, to evaluate the heart chamber specific characteristics of the generated CMs across 12 samples, we followed a chamber-specific marker approach, the ventricle specific gene *MYL2* was expressed below the expression threshold (NRC ≥ 20) in the majority of our samples. Lee et al. [22] have shown that induction of retinoic acid (RA) signaling in the early stages of iPSC-to-cardiomyocyte differentiation significantly down-regulated *MYL2* expression and promoted atrial fate specification of iPSC-differentiated CMs. A switch from *MYL7* to *MYL2* has also been shown to be associated with maturation of ventricular CMs. *MYL7* is expressed in all early fetal CMs and switches to *MYL2* as ventricular CMs mature and the *MYL7* expression becomes restricted to atrial cardiomyocytes [30,35,36]. *MYL7* was highly expressed in all 12 samples and was significantly upregulated during CMs differentiation (Table S1). The expression of ventricle-specific transcription factor *HEY2* did not change significantly between iPSCs and differentiated CMs and was moderately expressed (average NRC = 181.02) in all 12 samples. *HEY2* expression plays an important role in ventricular, especially left ventricular, development by suppressing atrial identity [28,37,38,39]. However, contrary to the previously reported reduced expression of *IRX4* and *MYH7* in RA-treated CMs [22], the expressions of these ventricle-associated genes were significantly upregulated (FC = 20.4 and 1331.1, respectively) in our iPSC-generated CMs, which may warrant further investigation. The expression of atrial-specific transcription factor *HEY1*, which plays an important role in the precise formation of the heart’s atrioventricular boundary [40,41,42], was significantly upregulated in iPSC-differentiated CMs (FC = 16.9), and its expression was about 2-fold higher (average NRC = 362) than that of *HEY2*. The expression levels of other atrial-specific genes (*NR2F2*, *TBX5*, *NPPA*, *MYL7*, *CACNA1D,* and *KCNA5)* and sino-atrial node associated genes (*TBX3*, *HCN4,* and *CACNA1G)* were also significantly upregulated (Table S1) [22,27,28].

Human ESC/iPSC-derived CMs exhibit immature phenotypes that resemble fetal cardiomyocytes, and despite tremendous progress in maturation methods, complete maturation of the ESC/iPSC-derived CMs is yet to be achieved [30,43,44,45,46,47,48]. However, for an epidemiological-scale disease gene discovery approach, obtaining primary cardiomyocytes from patients and healthy controls is nearly impossible; therefore, iPSC-derived CMs whose maturation state across samples are uniform/comparable holds tremendous potential as the closest surrogate of primary cardiomyocytes. As detailed in the results, we assessed the maturation state of our iPSC-generated CMs across 12 samples using quantitative expression of genes involved in CM maturation processes such as myofibril maturation, expression of ion channel handling genes, changes in the expression of genes involved in cellular metabolism, and changes in the expression of cell cycle and cell adhesion associated genes (Figure 2A). The sarcomeric isoforms of both mature and immature myofibrils marked by *MYH6*, *MYH7*, *TNNI1*, *TNNI3,* and *MYL7* were expressed and significantly upregulated in our iPSC-differentiated CMs; however, *MYL2* expression was below the expression threshold or borderline expressed in most samples. As indicated above, *MYL7* is predominantly expressed in fetal, as well as mature, atrial cardiomyocytes, and *MYL2* expression is restricted to mature ventricular cardiomyocytes [30]. It is also important to note that expression of all mature myofibril isoforms was lower than the immature myofibril isoforms (Figure 2A). The automaticity ion channel gene *HCN4* and calcium handling molecules *CACNA1C*, *RYR2*, and *ATP2A2* [30,49,50] were upregulated and moderately expressed in all 12 CM samples. However, the expression of mature ventricular ion channel *KCNJ2* was below the expression threshold or borderline expressed in most samples. The metabolic maturation of CMs involves activation of metabolic transcriptional regulators, upregulation of fatty acid metabolism, oxidative phosphorylation, and mitochondrial biogenesis genes, and downregulation of glycolytic genes [51,52,53]. The expression of metabolic transcriptional regulators *PPARGC1A* and *PPARA* were significantly upregulated in our iPSC-differentiated CMs. The metabolic transcriptional regulators *NRF1* and *ESRRA*, though expressed in all samples, did not change significantly in iPSCs and their differentiated CMs. Hexokinase, which executes the first step of glycolysis, is predominantly isoform *HK1* in immature (fetal and neonatal) CMs, and isoform *HK2*, which exhibits less glycolytic activity, is predominantly expressed in mature CMs. Both *HK1* and *HK2* were highly expressed in our iPSC-differentiated CMs (Figure 2A). However, *HK1* expression was nominally higher than *HK2* (FC = 1.5). Cardiomyocyte proliferation rate declines rapidly postnatally and upon maturation of the iPSC-differentiated CMs [30]. The cell cycle regulators *CDK1*, *CCNB1,* and *AURKB*, which promote G2/M phase progression during the cell cycle [54], were significantly down-regulated in our iPSC-differentiated CMs (Table S1). The co-overexpression of *CDK1-CCNB1* and *CDK4-CCND* complexes that are involved in G2/M phase and G1/S phase progressions, respectively, were sufficient to reactivate proliferation in mature, cell division ceased CMs [55]. The expressions of *CDK4*, *CCNDs,* and other activators of CM proliferation, the *ERBB2* and *YAP1,* did not change significantly between iPSCs and their differentiated CMs. Specialized cardiomyocyte–cardiomyocyte junctions, the intercalated discs (ICDs) are involved in maturational integration of CMs into cardiac tissue. Immature CMs lack ICDs, and ICD components are either not expressed, localized to the interior of cells, or are throughout the cell surface. ICDs are hybrid junctions and comprise three major types of cell adhesions: fascia adherens, desmosomes, and gap junctions [56,57]. The fascia adherens protein gene *CDH2*; desmosomes-associated genes *DSG2*, *DSC2*, *JUP*, *PKP2*, and *DSP*; and gap junction protein gene *GJA1* were all significantly upregulated in our iPSC-differentiated CMs (Figure 2A).

Overall, our iPSC-differentiated CMs have a transcriptomic and functional profile of immature atrial-like CMs. The correlation analysis based on CM-specific, CM-subtype-specific, and CM maturity-associated genes, however, suggests a highly concordant CM population across our 12 samples (Figure 2B–D).

Having comprehensively evaluated the transcriptomic and functional characteristics of our iPSC-differentiated CMs across 12 samples, we next explored the cardiomyopathies and associated heart disease modeling potential of the iPSC-generated CMs. The enrichment analysis of the CM upregulated transcriptome (2116 genes) in the ClinVar-2019 gene sets [58] showed significant enrichment of cardiomyopathies and associated disease. Nearly 70% to 80% of genes listed as associated with cardiomyopathies and associated disease in the ClinVar 2019 data set were significantly upregulated in our iPSC-differentiated CMs (Table 3). Furthermore, the CM-upregulated transcriptome also showed significantly high enrichment in a gene set (GSE150392) down-regulated by SARS-CoV-2 infection in cardiomyocytes. These results strongly suggest that our iPSC-generated CMs, that are highly concordant in their transcriptomic and functional characteristics across multiple samples, are a relevant cell model to study cardiomyopathy and associated heart disease. Furthermore, additional maturation and subtype-specific characterization of the generated CMs including the electrophysiological characterization may be performed on a small sample to validate the findings of a larger, epidemiological-scale disease gene discovery study or to model a well-characterized disease-specific phenotype.

### 3.2. MACE-Associated Transcriptomic Changes in Familial CM Samples

As detailed in the results, differential gene expression analysis of the CM-upregulated transcriptome (2116 genes) identified 179 genes that were significantly DE (moderated *t* statistics *p*-value ≤ 0.05 and FC-abs ≥ 2.0) between the MACE-affected and unaffected sibships. The disease and function annotation analysis of the 179 DE genes in IPA identified significant enrichment and predicted activation of arrhythmia (*p*-value = 1.19 × 10^−8^; activation *z*-score = 2.4). Furthermore, atrial fibrillation was also found significantly enriched using IPA as well as Jensen diseases data set (*p*-values = 1.06 × 10^−5^ and 5.53 × 10^−5^, respectively) [25]. An upstream regulator analysis of the 179 DE genes in IPA identified significant down-regulation of *PITX2* gene (FC = −2.6) in the MACE-affected sibship and dysregulated expression of its downstream targets (54 genes significantly DE between the MACE-affected and unaffected sibships), and several of them were enriched in arrhythmia (Figure 4A). The *PITX2*-associated variability including over- and under- expression were reported in atrial fibrillation patients, and a dose-dependent regulatory role of *PITX2* in normal atrial function has been suggested [59,60,61]. The causal role of *PITX2* variation in atrial fibrillation and overall arrhythmogenesis involves multiple regulatory pathways [62]. The *PITX2* forms an incoherent regulatory circuit with *TBX5* (significantly downregulated in the MACE-affected sibship; FC = −2.7); both regulate each other’s expression and also regulate the expression of their downstream targets antagonistically, and perturbation of this co-regulated network increases atrial fibrillation susceptibility [63,64,65]. The expressions of *PITX2* and *TBX5* downstream targets, the delayed rectifier potassium channel gene *KCNA5,* and the low-voltage-activated calcium channel gene *CACNA1G* were significantly downregulated in the MACE-affected sibship (FC = −3.0 and −3.2, respectively). The loss-of-function mutations and reduced expression of these ion channel genes were reported in atrial fibrillation patients [66,67,68,69,70,71]. The *PITX2* also antagonistically regulates *TBX3* expression [72,73], which was significantly upregulated (FC = 4.5) in the MACE-affected sibship. *TBX3*, another member of the T-box transcription factors, regulates the sino-atrial-node gene program, and its upregulated expression causes sino-atrial remodeling of mature atrial and ventricular cardiomyocytes including remodeling of the contractile apparatus, which is poorly developed in sino-atrial/pacemaker cells [74,75,76]. The expression of one of its components, the *TNNI3,* which is also a marker for CM maturation, was significantly downregulated (FC = 3.7) in the MACE-affected sibship. The expression of Z-discs and intercalated discs associated *DES* was also significantly down regulated in the MACE-affected sibship (Table S4). The down regulated expressions of *PITX2* and *TBX5,* and associated sino-atrial and fetal-like remodeling of cardiomyocytes, may also be involved in dysregulation of DE genes enriched in “abnormality of heart ventricle” and “dysfunction of heart”, for example, significant down-regulation of *HAND1* and *HAND2* (FC *=* −2.3 and −2.2, respectively) in the MACE-affected sibship. The expressions of *HAND1* and *HAND2* play a role, which is poorly understood, in left and right ventricle development and overall cardio morphogenesis [77,78]. The “blood clot” and “thrombus” formation were also significantly enriched and nominally activated (*p*-value = 8.05 × 10^−8^ and 2.99 × 10^−7^, respectively; activation *z*-score = 1.9 and 1.1, respectively) in the 179 DE genes, suggesting activation of coagulation and prothrombotic intrinsic program in CMs generated from the MACE-affected sibship. Aortic atherosclerosis was also predicted to be significantly activated (activation *z*-score = 2.2) in the MACE-affected sibship. There is contradictory evidence that atrial fibrillation and arrhythmia-induced remodeling of CMs may activate the blood coagulation and prothrombotic intrinsic program in atrial CMs [79,80,81,82,83]. The other arrhythmia-associated risk factors such as “fibrosis of myocardium” and “hypertension” were also significantly enriched (*p*-value = 4.06 × 10^−5^ and 6.14 × 10^−5^, respectively) in 179 genes found DE between the MACE-affected and unaffected sibships. Furthermore, the expression of *AGT* was significantly upregulated (FC = 3.6) in CMs generated from the MACE-affected sibship samples. The upregulated expression of *AGT* is a risk factor for several of the disease conditions enriched in the MACE-associated DE genes (Figure 4). The enrichment of genes found DE between MACE-affected sibship CMs and MACE-unaffected sibship CMs in atrial fibrillation/arrhythmia and prothrombotic and atherosclerosis disease conditions (Figure 4) correlates with risk favoring adverse cardiac events in the MACE-affected sibship [84,85]. Overall, our results show that human iPSC-derived CM cellular/gene expression phenotypes likely exist that correlate with genetic risk of MACE. This new finding supports larger epidemiological-scale studies of iPSC-generated CMs to identify quantitative cellular phenotypes that better index disease risk.

## 4. Materials and Methods

The 12 validated iPSC lines used in this study were previously reprogrammed from cryopreserved lymphoblastoid cells lines (LCLs) of our San Antonio Mexican American Family Study (SAMAFS) participants and following the methodology described in our previous publications [17,18].

### 4.1. CMs Differentiation

The differentiation of validated iPSC lines (Figure S1) into spontaneously contracting CMs was performed using the commercially available Gibco PSC Cardiomyocyte Differentiation Kit (Thermo Fisher Scientific, Waltham, MA, USA), and methods are summarized in the schematic presented in Figure 1A. Briefly, well-characterized and validated iPSCs cultured in feeder-free conditions until 70% to 80% confluent were harvested using Gibco StemPro Accutase and then seeded on Geltrex^TM^ (both from Thermo Fisher Scientific, Waltham, MA, USA) coated tissue culture plates at a density of ~4 × 10^4^ cells/cm^2^ in mTeSR-1 medium (Stem Cell Technologies Inc., Cambridge, MA, USA) supplemented with 10 µM ROCK inhibitor Y27632 (ATCC, Manassas, VA, USA). The mTeSR-1 medium was changed daily for the following two days or until the culture reached 50% to 70% confluency. On day 4, when iPSC cultures were 50% to 70% confluent, the medium was switched to Gibco Cardiomyocytes Differentiation Medium A. On day 6, the medium was replaced with Gibco Cardiomyocytes Differentiation Medium B, and from day 8 onwards, the differentiating cells were maintained in Gibco Cardiomyocyte Maintenance Medium until day 14, and the medium was changed every other day (the Gibco Cardiomyocytes Differentiation Medium A, Medium B, and the Gibco Cardiomyocyte Maintenance Medium were all part of the Gibco PSC Cardiomyocyte Differentiation Kit). On day 14, the 12 iPSC-differentiated, spontaneously contracting CM lines were harvested for characterization and total RNA extraction.

### 4.2. Characterization of Generated CMs

Each generated CM line was characterized by two complementary methods: (1) the ICC analysis of cardiac mesoderm marker NKX2-5 and mature cardiomyocyte marker TNNT2; and (2) the genome-wide mRNA sequencing-based gene expression analysis of the cardiomyocyte-specific genes/markers as detailed in Figure 1D and Figure S2.

For ICC characterization, each iPSC-differentiated CM line was fixed using 4% paraformaldehyde (MilliporeSigma, St. Louis, MO, USA) and then immunoassayed using commercially available primary antibodies against cardiac mesoderm marker NKX2-5 and cardiomyocyte marker TNNT2 (both from Thermo Fisher Scientific, Waltham, MA, USA) and standard ICC techniques. In each assay, cells were counterstained with 4’,6-diamidino-2-phenylindole dihydrochloride (DAPI) nuclear stain (MilliporeSigma, St. Louis, MO, USA) and were imaged on a Carl Zeiss epifluorescence equipped inverted microscope (Carl Zeiss Microscopy, LLC, White Plains, NY, USA).

### 4.3. RNA Extraction and Sequencing

Total RNA extraction: The total RNA was extracted from snap-frozen cell pellets (~3–5 × 10^6^ cells) of 12 well-characterized, validated iPSC lines and their differentiated CM lines using the commercially available RNeasy Mini Kit (Qiagen, Germantown, MD, USA) and the manufacturer’s protocol. RNA quality and quantity were assessed using a NanoDrop 2000 Spectrophotometer (Thermo Fisher Scientific, Waltham, MA, USA) and an Agilent 2200 TapeStation system (Agilent, Santa Clara, CA, USA).

mRNA Sequencing: The mRNA sequencing libraries were prepared using 1µg total RNA per sample (extracted from 12 iPSC and 12 CM lines) and the Illumina TruSeq RNA sample preparation kit v2 (Illumina, Inc., San Diego, CA, USA). Briefly, the poly-A tail containing mRNA molecules was enriched from total RNA using oligo dT attached magnetic beads. The mRNA-enriched samples were then fragmented into ∼200–600 base pair sizes using divalent cations and elevated temperature. The first-strand cDNA was synthesized from resulting cleaved RNA fragments and using reverse transcriptase, and random primers, followed by second-strand cDNA synthesis, using DNA polymerase-I and RNase H. The synthesized cDNA fragments were end-repaired, and adaptor ligations were performed. The end-repaired and adaptor ligated cDNA libraries were purified and enriched by PCR and then deep-sequenced on an Illumina HiSeq 2500 platform.

Sequence Analysis: Raw fastq sequence files were generated and demultiplexed using Illumina bcl2fastq software (Illumina, Inc., San Diego, CA, USA). After pre-alignment QCs, sequences were aligned to human genome assembly GRCh38 (hg38) and mapped to RefSeq transcripts using StrandNGS software v3.4 (Strand Life Sciences Pvt. Ltd., Bangalore, India). The aligned reads were filtered based on default read quality metrics, and log transformation and “DESeq” normalization were applied. The known mRNAs having NRC ≥ 20 in 12 iPSC and/or their differentiated CM lines were considered expressed and selected for differential gene expression analysis. For CMs, expressed transcriptome genes/mRNAs having NRC ≥ 20 in all 12 CM lines were counted expressed.

### 4.4. Differential Gene Expression Analyses

To identify genes/mRNAs that were DE between iPSCs and their differentiated CMs and between MACE-affected and unaffected sibships, moderated *t* statistics and expression fold change analyses were performed. The significance criteria for each differential gene expression analysis are detailed in the results.

### 4.5. Disease and Function Annotation Analyses

Disease and functional annotations and upstream regulator analyses were performed on significantly DE genes/mRNAs identified between iPSCs and their differentiated CMs, and between MACE-affected and unaffected sibships using the IPA platform (QIAGEN Digital Insights, Redwood City, CA, USA) and Enrichr, a gene list enrichment analysis web tool [23,24]. In IPA, right-tailed Fisher’s exact test *p*-values corrected for FDR were used to calculate enrichment significance, and the direction of functional change was assessed by the activation *z*-score detailed in [86]. In Enrichr, enrichment significance was computed using three approaches detailed in Chen et al. [23]. The first one is the Fisher exact test, and we have ranked our Enrichr results presented in Table 1, Table 2, and Table 3 based on Fisher exact test *p*-values. We have also presented in Table 1, Table 2, and Table 3 the adjusted enrichment *p*-values computed in Enrichr by combining the three approaches detailed in [23,24].

## 5. Conclusions

In conclusion, we have demonstrated robust differentiation of spontaneously contracting CMs across multiple iPSC lines reprogrammed from cryopreserved LCLs. Our comprehensive transcriptomic analysis of the generated CM lines identified 4191 genes that were significantly DE between iPSCs and their differentiated CMs. The enrichment analysis of the CMs significantly upregulated transcriptome (2116 genes) in primary cell and tissue-specific gene sets shows that the generated CMs express CM-specific genes, and the upregulated transcriptome was significantly overlapped and enriched in primary cardiomyocytes and primary heart tissue upregulated genes. The transcriptomic analysis of the CM subtypes and maturation state shows that our iPSC-generated CMs possess a transcriptomic and functional profile of immature atrial-like CMs. However, the generated CM population across 12 samples was highly concordant, and disease and function enrichment analysis shows that the generated CMs are a relevant cell model to study cardiomyopathies and associated heart diseases. Furthermore, modeling the effect of MACE in our generated CM familial samples identified gene expression phenotypes consistent with the predisposition of the MACE-affected sibship to arrhythmia, prothrombotic, and atherosclerosis risk. It is important to note that MACE status was assigned after a subject suffered a clinically diagnosed adverse cardiac event in our longitudinal study.

Overall, our data and methodology provide a proof of the principle that iPSC-derived CMs, whose production is scalable to a large sample size for an epidemiological-scale disease gene discovery approach while maintaining uniform/comparable CM subpopulations and maturation states across samples, hold a tremendous potential as the closest surrogate of primary cardiomyocytes. Furthermore, our study shows that human iPSC-generated CMs express phenotypic variation that is correlated with genetic risk of MACE. The potential existence of novel cellular biomarkers supports larger studies of human iPSC-derived CMs. Such non-invasive cell-specific biomarkers may lead to improvements in the prediction of risk (even in children) and point to potential causal genes involved in disease risk.

## Figures and Tables

**Table 1:** Top 10 significantly enriched gene ontology biological processes in significantly upregulated CMs transcriptome.

GO Biological Process	Overlap	*p*-Value	Adjusted *p*-Value
Heart development (GO:0007507)	66/149	4.09 × 10^−26^	2.09 × 10^−22^
Extracellular matrix organization (GO:0030198)	75/229	6.62 × 10^−20^	1.69 × 10^−16^
Cardiac muscle tissue morphogenesis (GO:0055008)	27/36	1.43 × 10^−19^	2.43 × 10^−16^
Circulatory system development (GO:0072359)	48/109	3.09 × 10^−19^	3.94 × 10^−16^
Ventricular cardiac muscle tissue morphogenesis (GO:0055010)	24/34	1.56 × 10^−16^	1.60 × 10^−13^
Muscle contraction (GO:0006936)	49/137	3.45 × 10^−15^	2.94 × 10^−12^
Myofibril assembly (GO:0030239)	26/47	5.10 × 10^−14^	3.72 × 10^−11^
Cardiac ventricle morphogenesis (GO:0003208)	24/43	3.69 × 10^−13^	2.35 × 10^−10^
Regulation of heart contraction (GO:0008016)	36/95	2.15 × 10^−12^	1.22 × 10^−9^
cardiac atrium morphogenesis (GO:0003209)	11/17	1.22 × 10^−7^	1.27 × 10^−5^

Note: The gene ontology (GO) biological process annotation 2018 was used in the enrichment analysis.

**Table 2:** Top 5 significantly enriched human gene atlas cell types and GTEx tissue samples in significantly upregulated CMs transcriptome.

Human Tissue/Cell Types	Overlap	*p*-Value	Adjusted *p*-Value
**Human Gene Atlas** (up-regulated genes in human tissues from BioGPS)			
Uterus	94/405	5.11 × 10^−13^	2.15 × 10^−11^
Placenta	58/201	1.17 × 10^−13^	9.85 × 10^−12^
Heart	88/415	1.23 × 10^−10^	3.43 × 10^−9^
Smooth Muscle	78/363	7.28 × 10^−10^	1.53 × 10^−8^
Cardiac Myocytes	62/273	4.36 × 10^−9^	7.33 × 10^−8^
**GTEx Tissue Samples** (up-regulated genes)			
GTEX-X4EP-0326-SM-3P5Z6 heart female 60–69 years	210/755	1.37 × 10^−41^	4.00 × 10^−38^
GTEX-X62O-0826-SM-46MW8 heart male 50–59 years	208/766	1.79 × 10^−39^	2.62 × 10^−36^
GTEX-SE5C-0626-SM-2XCDV heart female 40–49 years	229/906	4.75 × 10^−38^	4.62 × 10^−35^
GTEX-S3XE-0426-SM-3K2AC heart male 50–59 years	163/547	2.78 × 10^−36^	2.03 × 10^−33^
GTEX-WY7C-1126-SM-3NB3A heart male 50–59 years	176/637	1.88 × 10^−34^	1.09 × 10^−31^

Note: Enrichment analysis into human gene atlas and Genotype-Tissue Expression (GTEx) project tissue sample gene sets were performed in Enrichr [23,24], and gene sets were accessed on November 2020.

**Table 3:** Top 5 significantly enriched ClinVar (2019) and one COVID-19-related gene set in significantly upregulated CMs transcriptome.

Diseases	Overlap	*p*-Value	Adjusted *p*-Value
**ClinVar (2019) Gene Sets**			
Cardiomyopathy	33/48	1.13 × 10^−21^	2.06 × 10^−19^
Primary dilated cardiomyopathy	23/34	2.94 × 10^−15^	2.67 × 10^−13^
Primary familial hypertrophic cardiomyopathy	18/22	1.24 × 10^−14^	7.54 × 10^−13^
Familial dilated cardiomyopathy	21/30	1.66 × 10^−14^	7.53 × 10^−13^
Myofibrillar myopathy	5/6	7.22 × 10^−5^	2.19 × 10^−3^
**COVID-19 Related Cardiomyocyte Gene Set**			
Down-regulated by SARS-CoV-2 in cardiomyocytes from GSE150392	143/499	6.53 × 10^−30^	1.34 × 10^−27^

Notes: (1) ClinVar 2019 disease gene set was used in this enrichment analysis. (2) Only one SARS-CoV-2 (COVID-19)-related cardiomyocytes gene set was available at the time of this analysis.

## Data Availability

The mRNA sequence data generated from the 12 iPSC lines and their 12 differentiated CM lines were submitted to the gene expression omnibus (GEO) archive and are available under the accession number GSE165242.

## Supplementary Materials

The following are available online at https://www.mdpi.com/1422-0067/22/7/3311/s1.
