# Supplementary material for: Disease Modeling and Disease Gene Discovery in Cardiomyopathies: A Molecular Study of Induced Pluripotent Stem Cell Generated Cardiomyocytes
Source: Int J Mol Sci. 2021 Mar 24;22(7):3311. doi: 10.3390/ijms22073311 (PMC8037452; doi:10.3390/ijms22073311)
Supplement: Supplementary file 1 [file ijms-22-03311-s001.zip › Figure S1.pdf]

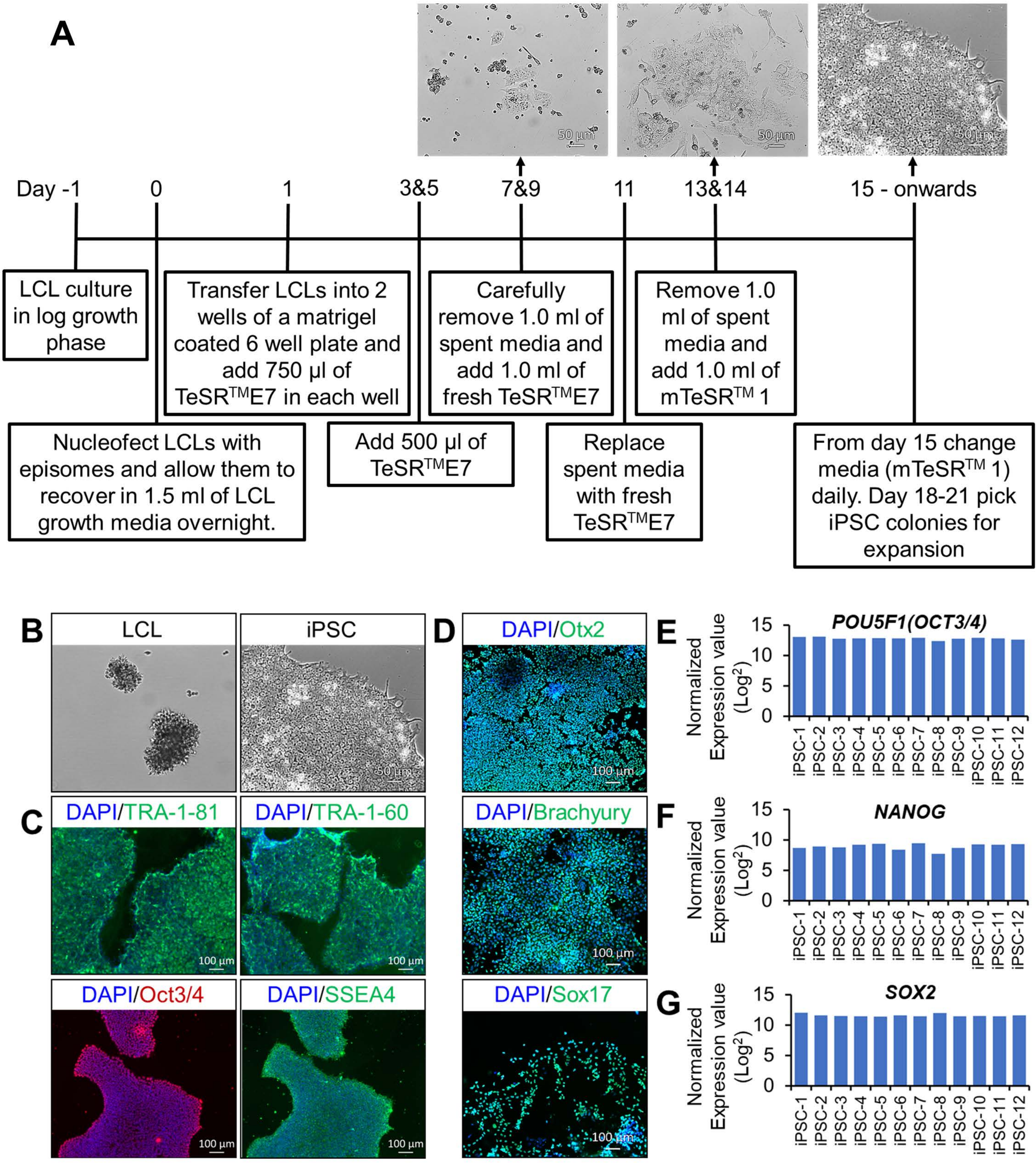

Figure S1. iPSC reprogramming and validation strategy. (A) Schematic diagram outlining the iPSC reprogramming protocol. (B) Bright filed images of LCLs and reprogrammed iPSCs. (C) Immunocytochemistry (ICC) analysis of the generated iPSCs showing expression of the pluripotency markers. (D) ICC analysis of the cells of all three embryonic germ layers differentiated from the reprogrammed iPSCs. (E-G) Bar-graphs showing normalized (Log^2) gene expression values of iPSC core pluripotency markers/genes in all the 12 reprogrammed iPSCs.
