# Supplementary material for: Disease Modeling and Disease Gene Discovery in Cardiomyopathies: A Molecular Study of Induced Pluripotent Stem Cell Generated Cardiomyocytes
Source: Int J Mol Sci. 2021 Mar 24;22(7):3311. doi: 10.3390/ijms22073311 (PMC8037452; doi:10.3390/ijms22073311)
Supplement: Supplementary file 1 [file ijms-22-03311-s001.zip › Figure S2.pdf]

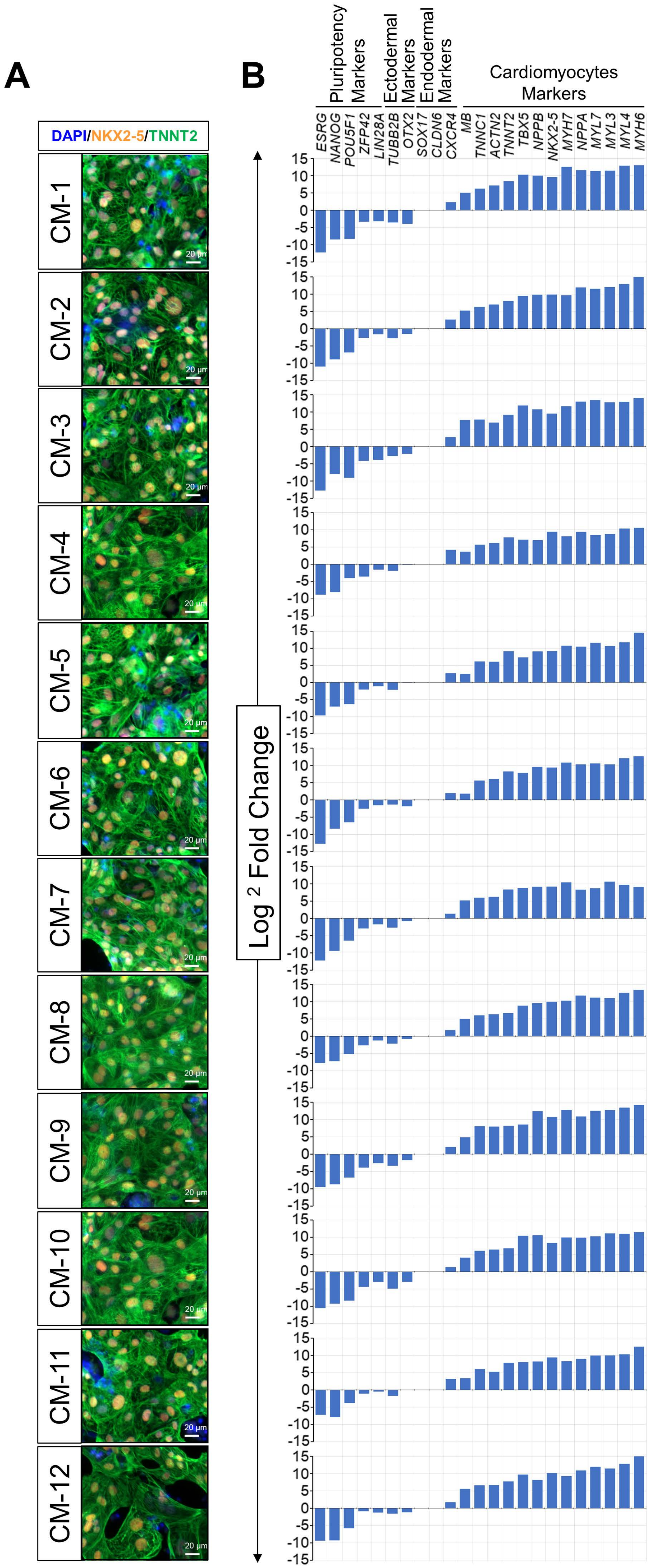

Figure S2. Characterization of all 12 iPSC generated CM lines by ICC and gene expression analysis. (A) ICC image panel showing expression of the CM specific markers NKX2-5 and TNNT2 in all 12 iPSC generated CM lines. (B) Gene expression graphs showing differential expression of cardiomyocytes, pluripotency, ectodermal and endodermal markers between 12 iPSCs and their generated 12 CM lines.
