# Supplementary material for: Disease Modeling and Disease Gene Discovery in Cardiomyopathies: A Molecular Study of Induced Pluripotent Stem Cell Generated Cardiomyocytes
Source: Int J Mol Sci. 2021 Mar 24;22(7):3311. doi: 10.3390/ijms22073311 (PMC8037452; doi:10.3390/ijms22073311)
Supplement: Supplementary file 1 [file ijms-22-03311-s001.zip › Table S2.docx]

Table S2: Enrichment *p*-values and activation *z*-score of cellular functions found significantly enriched in 2,075 genes that were significantly down regulated between iPSCs and their differentiated CMs.

| **Cellular Functions** | **Enrichment *p*-value** | **Predicted Activation State** | **Activation *z*-score** |
| --- | --- | --- | --- |
| ***Cell Death and Survival*** | | | |
| Cell survival | 1.13x10^-17^ | Decreased | -11.85 |
| Cell viability  Cell viability of tumor cell lines | 6.31x10^-19^  1.47x10^-14^ | Decreased  Decreased | -11.50  -10.29 |
| Cell viability of leukemia cell lines | 2.74x10^-08^ | Decreased | -4.11 |
| ***Cell Cycle*** | | | |
| Cell cycle progression | 3.77x10^-19^ | Decreased | -3.82 |
| M phase | 2.91E-10 | Decreased | -3.08 |
| Interphase | 3.25E-11 | Decreased | -3.03 |
| M phase of tumor cell lines | 1.03E-09 | Decreased | -2.76 |
| Mitosis | 2.31E-17 | Decreased | -2.74 |
| Segregation of chromosomes | 8.94E-20 | Decreased | -2.60 |
| G1/S phase transition | 5.68E-11 | Decreased | -2.39 |
| Checkpoint control | 2.28E-11 | Decreased | -2.27 |
| Interphase of tumor cell lines | 3.15E-07 | Decreased | -2.24 |
| ***Cellular Assembly and Organization*** | | | |
| Organization of cytoplasm | 5.08E-11 | Decreased | -6.33 |
| Organization of cytoskeleton | 3.31E-12 | Decreased | -6.28 |
| Microtubule dynamics | 2.37E-11 | Decreased | -6.17 |
| Alignment of chromosomes | 2.06E-10 | Decreased | -2.70 |
| Segregation of chromosomes | 8.94E-20 | Decreased | -2.60 |
| ***DNA Replication, Recombination, and Repair*** | | | |
| Synthesis of DNA | 3.96E-07 | Decreased | -3.95 |
| DNA replication | 1.16E-19 | Decreased | -3.08 |
| Metabolism of DNA | 3.10E-15 | Decreased | -2.92 |
| Alignment of chromosomes | 2.06E-10 | Decreased | -2.70 |
| Segregation of chromosomes | 8.94E-20 | Decreased | -2.60 |
| Checkpoint control | 2.28E-11 | Decreased | -2.27 |
