# Supplementary material for: Disease Modeling and Disease Gene Discovery in Cardiomyopathies: A Molecular Study of Induced Pluripotent Stem Cell Generated Cardiomyocytes
Source: Int J Mol Sci. 2021 Mar 24;22(7):3311. doi: 10.3390/ijms22073311 (PMC8037452; doi:10.3390/ijms22073311)
Supplement: Supplementary file 1 [file ijms-22-03311-s001.zip › Table S3.docx]

Table S3: Enrichment *p*-values and activation *z*-score of developmental and cellular functions found significantly enriched in 2,116 genes that were significantly up regulated between iPSCs and their differentiated CMs.

| **Developmental and Cellular Functions** | **Enrichment *p*-value** | **Predicted Activation State** | **Activation z-score** |
| --- | --- | --- | --- |
| ***Cardiovascular System Development and Function, Tissue Development*** | | | |
| Development of vasculature | 3.49E-52 | Increased | 6.444 |
| Angiogenesis | 9.75E-49 | Increased | 6.402 |
| Vasculogenesis | 3.62E-46 | Increased | 5.714 |
| Migration of endothelial cells | 8.95E-26 | Increased | 5.548 |
| Cell movement of endothelial cells | 1.72E-26 | Increased | 5.375 |
| Contractility of cardiac muscle | 1.12E-21 | Increased | 4.973 |
| Function of cardiac muscle | 2.20E-24 | Increased | 4.869 |
| Cardiac contractility | 3.71E-26 | Increased | 4.56 |
| Vascularization | 2.82E-18 | Increased | 4.471 |
| Cardiogenesis | 1.06E-56 | Increased | 4.106 |
| Tubulation of endothelial cells | 1.39E-14 | Increased | 3.468 |
| Formation of myofibrils | 2.91E-17 | Increased | 3.388 |
| Heart rate | 3.41E-29 | Increased | 3.278 |
| Morphogenesis of cardiovascular system | 1.42E-51 | Increased | 3.231 |
| Morphogenesis of heart | 5.43E-43 | Increased | 2.425 |
| Endothelial cell development | 1.10E-18 | Increased | 2.074 |
| Development of cardiovascular tissue | 4.61E-20 | Increased | 1.992 |
